# Changes in university students cognitive-linguistic performance following a structured didactic intervention

**DOI:** 10.3389/fpsyg.2026.1779389

**Published:** 2026-09-03

**Authors:** Teófilo Félix Valentín Melgarejo, Fidel Alberto García Yale, Gastón Jeremías Oscátegui Nájera, Clodoaldo Ramos Pando, Víctor Luis Albornoz Dávila, Raúl Malpartida Lovatón, William César Santos Hinostroza, Susy Rosy Santiago Lázaro, Liz Ketty Bernaldo Faustino, Lilia Mariela Matos Atanacio, Isabel Alejandrina Delzo Calderón, Pablo Lolo Valentín Melgarejo

**Affiliations:** 1 Program of Communication and Literature, Faculty of Education Sciences, Universidad Nacional Daniel Alcides Carrión, Cerro de Pasco, Peru; 2 Faculty of Education Sciences, School of Primary Education, Universidad Nacional Hermilio Valdizán, Huánuco, Peru; 3 Faculty of Education Sciences, School of Primary Education, Universidad Nacional Daniel Alcides Carrión, Cerro de Pasco, Peru; 4 Program of Mathematics and Physics, Faculty of Education Sciences, Universidad Nacional Daniel Alcides Carrión, Cerro de Pasco, Peru; 5 Program of History, Social Sciences and Tourism, Faculty of Education Sciences, Universidad Nacional Daniel Alcides Carrión, Cerro de Pasco, Peru; 6 Program of Biology and Chemistry, Faculty of Education Sciences, Universidad Nacional Daniel Alcides Carrión, Cerro de Pasco, Peru

**Keywords:** cognitive-linguistic performance, educational intervention, executive functions, language processing, oral expression, reading comprehension, written expression

## Abstract

**Background:**

Cognitive-linguistic functioning involves the coordinated operation of executive control, language processing, working memory, and semantic organization, which are important for academic performance. Although structured educational interventions can improve specific learning outcomes, relatively few studies have examined whether such interventions are associated with coordinated changes across oral expression, written expression, and reading comprehension.

**Objective:**

This study examined changes in global cognitive-linguistic performance and its three assessed domains oral expression, written expression, and reading comprehension following a structured didactic intervention among university students.

**Methods:**

A prospective one-group quasi-experimental pre–post design was used. Twenty-six undergraduate students completed a 6-week intervention comprising 18 structured sessions that integrated cognitive activation, language-focused activities, guided practice, feedback, and metacognitive reflection. Cognitive-linguistic performance was assessed immediately before and after the intervention using a researcher-developed battery. Paired-samples tests, Holm-adjusted *p*-values, mean differences with 95% confidence intervals, within-participant effect sizes, and performance-level transitions were examined.

**Results:**

Global cognitive-linguistic performance increased from 45.35 ± 4.38 at baseline to 56.88 ± 5.54 after the intervention, corresponding to a mean change of 11.53 points (95% CI: 9.64–13.42; Holm-adjusted *p* < 0.001; Cohen’s *d*_z_ = 2.46). Improvements were also observed in oral expression (mean change = 4.19; 95% CI: 3.26–5.12; *d*_z_ = 1.82), written expression (mean change = 3.72; 95% CI: 2.79–4.65; *d*_z_ = 1.61), and reading comprehension (mean change = 3.62; 95% CI: 2.65–4.59; *d*_z_ = 1.50); all Holm-adjusted *p*-values were <0.001. Twenty-one participants moved to a higher performance category, five remained in the same category, and none moved to a lower category.

**Conclusion:**

Participation in the structured didactic intervention was associated with substantial improvements in global cognitive-linguistic performance and across all three assessed domains. The multidomain pattern of improvement and high implementation fidelity supports the feasibility of integrating cognitive, linguistic, and metacognitive activities within university instruction. However, the small sample, one-group design, and absence of delayed follow-up prevent definitive causal conclusions and do not establish generalized or durable cognitive transfer.

## Introduction

Cognitive-linguistic competence is one of the key foundations of academic functioning and involves the coordinated action of executive control, language processing, working memory, and semantic integration. These processes support advanced academic activities, including reading comprehension, written expression, verbal reasoning, information synthesis, and metacognitive regulation. Rather than functioning as completely independent abilities, executive and linguistic processes interact when students maintain task goals, organize information, retrieve relevant knowledge, suppress irrelevant responses, and monitor the quality of their performance (Diamond, 2013; Ellis, 2019; Miyake and Friedman, 2012).

In university education, students are expected to participate in cognitively demanding activities such as interpreting complex texts, constructing evidence-based arguments, explaining academic concepts, and integrating information from different sources. Successful performance on these tasks depends not only on subject-specific knowledge but also on attentional control, working-memory updating, semantic organization, planning, and the adaptive use of language. Individual variation in these processes may consequently contribute to differences in students’ academic-language performance (Castillo et al., 2022; Long et al., 2020; Feryok, 2010).

Despite this increased awareness, educational research has frequently examined reading, writing, and oral expression as separate outcomes without fully considering their shared executive and semantic foundations. Similarly, many instructional interventions have aimed to improve discrete skills rather than investigating whether a structured program is associated with coordinated changes across several communicative domains. Models of language processing, however, indicate that oral production, written production, and reading comprehension rely partly on overlapping mental resources, including working memory, attentional control, lexical access, planning, and semantic integration (Chomsky, 2011; Ellis, 2019; Kellogg, 2008; Perfetti and Stafura, 2014).

Structured didactic interventions may provide opportunities to strengthen the application of these processes within authentic academic-language tasks. Explicit strategy modeling, guided practice, progressively demanding activities, criteria-based feedback, and metacognitive reflection can encourage students to plan, monitor, evaluate, and revise their performance. Nevertheless, improvement on trained or closely related tasks should not automatically be interpreted as generalized cognitive enhancement. Evidence regarding transfer from cognitive training to broader, untrained abilities remains mixed, and outcomes may depend on task similarity, intervention duration, implementation quality, participant expectations, and study design (de Boer et al., 2014; Diamond and Ling, 2016; Melby-Lervåg et al., 2016).

The present study focused on oral expression, written expression, and reading comprehension because these domains represent productive and text-based academic-language abilities that could be assessed under standardized administration and scoring conditions within the predefined intervention framework. Listening comprehension was not included because its assessment would have required separately developed and controlled audio materials, administration procedures, and scoring specifications beyond the scope of the present study. This delimitation does not imply that listening is unimportant to cognitive-linguistic functioning. Accordingly, the present findings should not be generalized to listening comprehension, which should be investigated in future extensions of the intervention.

A further concern in educational intervention research is that studies frequently report changes in mean scores without adequately describing intervention delivery, participant exposure, measurement quality, effect-size precision, or movement between educational performance categories. Furthermore, a one-group pre–post design cannot distinguish intervention-associated change from alternative explanations such as repeated testing, maturation, concurrent coursework, expectancy effects, or regression to the mean. Transparent reporting of these design limitations is therefore necessary to avoid overstating the effectiveness or transferability of an intervention.

Given the importance of executive control and language-related processes in university learning, the present study evaluated changes in undergraduate students’ cognitive-linguistic performance following a 6-week structured didactic intervention. The program combined cognitive activation, oral expression, written expression, reading comprehension, integrated language practice, feedback, and metacognitive reflection. The study examined changes in the global cognitive-linguistic score, changes within each assessed domain, and transitions among predefined educational performance categories. Because the study employed a one-group pre–post design, its purpose was to estimate within-participant change and evaluate the feasibility of the instructional approach rather than establish definitive causal effectiveness.

## Literature review

### Executive control and academic-language performance

Executive functions are a set of co-related cognitive processes that facilitate goal-directed behavior that involve working-memory updating, inhibitory control, cognitive flexibility, planning and self-monitoring (Diamond, 2013; Miyake and Friedman, 2012). In an academic context, such processes allow students to hold information and suppress alternative responses, switch back and forth between task demands, plan an action series, and assess their performance against the desired goal. Executive control is, therefore, not distinct from the processes of language use, but it is a part of planning, producing, revising, and comprehending extended discourse.

Executive functioning has been characterized by both shared and process-specific elements (Miyake and Friedman, 2012). This distinction is of importance for cognitive-linguistic performance. Attention, working memory, planning, and monitoring processes can be shared among the three expression modalities (oral, written, or reading), and the demands for domain-specific processes are the same across the three modalities. Therefore, gains in multiple language tasks may be due to multiple repeated regulatory strategies, but not necessarily to a common mechanism or to a generalized improvement of executive functioning.

### Working memory and language processing

Working memory is a temporary representational workspace for the storage and manipulation of linguistic information. Oral expressions require students to remember the intended message and to choose words, arrange propositions, control flow of ideas and fine-tune the message. For written expressions, there is the need to coordinate the production of content, its syntactic formulation, its transcription, its evaluation and revision. Structured practice can be used to help students to cope with the demands of the processes of writing when they have limited cognitive resources to devote to them (Kellogg, 2008).

Reading comprehension also involves having to hold information from an earlier part of the text in memory, link it to the rest of the text, make inferences, and keep track of the overall coherence of the evolving interpretation. Word knowledge and integration processes have been mentioned as important for building a meaningful representation of a text by Perfetti and Stafura (2014). Oral expression, writing, and reading can then be regarded as distinct academic outcomes, but with some of the same executive and language-processing resources (Chodkiewicz and Boyle, 2017).

Impairments in working memory do not imply that interventions in training any one cognitive function will generally lead to widespread gains in all the school’s domains of learning. Melby-Lervåg et al. (2016) reported that there was consistent evidence of far transfer to trained or related tasks, but little to no evidence of robust far transfer to intelligence or other tasks that do not share a close connection with the trained tasks. This separation of improvement for related tasks and generalized transfer is significant when interventions and assessments are conceptually linked.

### Semantic integration across communicative domains

Semantic integration is the process whereby the meanings of the words, sentence information, prior knowledge, and context cues are all integrated into a coherent representation. Important for recognizing relationships between ideas, for drawing inferences, for structuring a text, and for crafting effective written responses. Academic achievement requires their integration with the task of coordinating executive control, working memory, lexical access, syntactic processing and semantic integration rather than with one another working independently (Kintsch, 2018).

Semantic integration helps to build a coherent interpretation between sentences and paragraphs in reading. In writing, it helps the student to make claims, provide evidence and make explanations within an organized response. In oral expression, it enables the selection and sequencing of information and ensures coherence. The three domains are examined in the same intervention, but outcome scores are maintained separately for each domain, and these demands are common, thus justifying the theoretical approach to studying the three domains in the same intervention (Hagoort et al., 2009).

### Evidence from instructional interventions

Previous intervention research provides encouraging but mixed evidence. Some studies suggest that cognitively demanding and language-focused instruction can improve trained academic skills. Rogowsky et al. (2013), for example, reported improvements in reading and writing following cognitive and linguistic skills training among college students. Meta-analytic evidence also indicates that instructional duration, organization, implementation quality, and alignment between instruction and assessment can influence the magnitude of educational outcomes (de Boer et al., 2014). These findings support the use of structured and sustained practice but do not establish that every intervention produces broad cognitive transfer.

Cognitive linguistics-inspired pedagogies may help learners recognize conceptual relationships and apply language strategies more deliberately. However, Liu and Qin (2024) reported variation in intervention approaches, methodological quality, and outcomes, indicating that conclusions depend on how cognitive-linguistic principles are operationalized. Similarly, reflective use of self-regulated learning strategies may increase students’ awareness of how they plan, monitor, and evaluate task performance (Kuiper, 2002). Metacognitive reflection may therefore complement language practice, although its contribution must be measured directly before it can be identified as an intervention mechanism.

Other research counsels against interpreting pre–post improvement as evidence of broad cognitive reorganization. Diamond and Ling (2016) emphasized that executive-function interventions differ considerably in their effectiveness and that apparent improvement may reflect task-specific practice, expectancy effects, implementation differences, or weaknesses in study design. Melby-Lervåg et al. (2016) similarly found limited support for far transfer from working-memory training. Accordingly, changes in academic-language tasks should be interpreted as improvements in the assessed competencies unless independent measures demonstrate transfer to broader executive or cognitive abilities.

### Research gap and study contribution

Three limitations remain evident in the literature. First, oral expression, written expression, and reading comprehension are frequently investigated separately despite their partly shared dependence on executive control, working memory, and semantic integration. Second, intervention activities and implementation procedures are often described too briefly to permit replication. Third, studies without active control groups cannot clearly distinguish intervention-associated change from testing effects, maturation, concurrent instruction, expectancy effects, or regression to the mean.

The present exploratory study addresses the first two limitations by examining changes across three academic-language domains and by documenting the structure, activities, attendance, and implementation fidelity of a 6-week didactic intervention. It does not resolve the third limitation because no control group was included. The findings are therefore interpreted as within-participant changes associated with participation in the program rather than definitive evidence that the intervention alone caused the observed outcomes.

### Study hypotheses

Based on the theoretical framework and previous evidence, the following directional hypotheses were formulated:

*H1*: Mean global cognitive-linguistic performance will be significantly higher at post-intervention assessment than at baseline.

*H2*: Mean oral-expression performance will be significantly higher at post-intervention assessment than at baseline.

*H3*: Mean written-expression performance will be significantly higher at post-intervention assessment than at baseline.

*H4*: Mean reading-comprehension performance will be significantly higher at post-intervention assessment than at baseline.

*H5*: The distribution of participants across the predefined educational performance categories will shift upward from baseline to post-intervention assessment, with a greater proportion of participants classified in the high-performance category after the intervention.

## Materials and methods

### Study design

This study used a prospective one-group quasi-experimental pre–post design to examine changes in university students’ cognitive-linguistic performance following a structured instructional program. The same participants were assessed before and immediately after the intervention. Because no comparison group was included, the study evaluated within-participant change but could not exclude alternative explanations such as repeated testing, maturation, concurrent coursework, expectancy effects, or regression to the mean.

### Participants

Participants were recruited through classroom announcements from the Communication and Literature Program at Universidad Nacional Daniel Alcides Carrión, Peru, between March and April 2025. Spanish was the language of instruction in the participating program and the language used for the intervention and all assessments. Eligibility criteria were current enrolment in the program, proficiency in Spanish, availability to complete the intervention and both assessments, and provision of written informed consent. Spanish-language proficiency was established through participants’ enrolment and regular academic participation in the Spanish-medium program; no separate standardized language-proficiency test was administered. Students were excluded from the paired analysis if they attended fewer than 15 of the 18 sessions, withdrew from the study, or did not complete either assessment.

**Figure 1:**
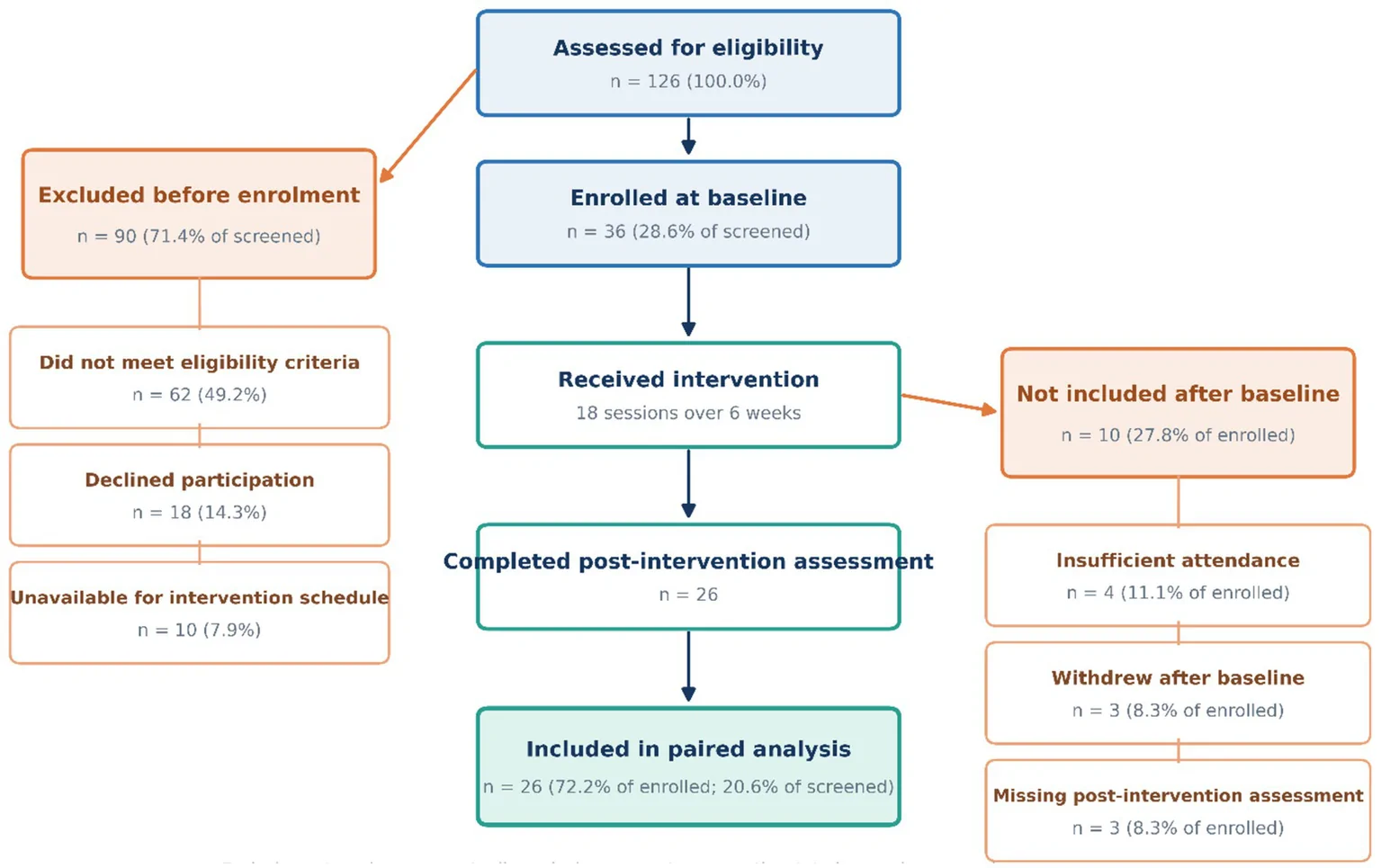
Participant recruitment, retention, and final analytical sample. Exclusion categories were mutually exclusive; percentages use the stated screening or enrolment denominator.

Of 126 students assessed for eligibility, 62 did not meet the eligibility criteria, 18 declined participations, and 10 were unavailable for the complete intervention schedule. Thirty-six students entered the study. Four were excluded because of insufficient attendance, three withdrew, and three did not complete the post-intervention assessment. The final analysis therefore included 26 participants (Figure 1).

The analyzed sample comprised 15 women and 11 men, with a mean age of 21.2 ± 1.8 years and an age range of 18–25 years. Fourteen participants were in their third academic year, and 12 were in their fourth year. Participation was voluntary, did not contribute to academic grades or course credit, and could be discontinued without academic consequences. Information about neurological, psychiatric, or diagnosed learning conditions was obtained through self-report; no clinical diagnostic assessment was conducted.

The sample was determined by the number of eligible students who completed the intervention and both assessments. No *a priori* power-based sample-size calculation was conducted. Therefore, the estimates are accompanied by confidence intervals and should be interpreted as preliminary.

### Instructional intervention

The intervention was implemented from 21 April to 30 May 2025 and comprised 18 sessions delivered over six consecutive weeks. Three 90-min sessions were conducted each week, corresponding to 27 instructional hours. The program integrated cognitive activation, oral expression, written expression, reading comprehension, integrated language practice, and metacognitive reflection (Kuiper, 2002; Liu and Qin, 2024). A complete example of one 90-min intervention session is provided in Supplementary Table S1.

The weekly sequence progressed from cognitive activation to domain-focused and integrated activities. Week 1 introduced attention, sequencing, working-memory, inhibition, and planning strategies. Week 2 focused on oral planning, fluency, organization, and monitoring. Week 3 addressed written planning, cohesion, syntax, and revision. Week 4 emphasized identification of central ideas, inference generation, semantic integration, and comprehension monitoring. Week 5 combined reading, discussion, oral interpretation, and written synthesis. Week 6 consolidated the strategies through cumulative tasks, peer review, self-evaluation, and planning for transfer to other academic activities.

Each session followed a standardized 90-min structure: cognitive activation (10 min), strategy introduction and modeling (15 min), guided domain-focused practice (25 min), integrated independent practice (25 min), feedback and correction (10 min), and metacognitive reflection (5 min). Participants used a short reflection form to identify the strategies applied, evaluate their effectiveness, and consider their use in other academic tasks.

**Figure 2:**
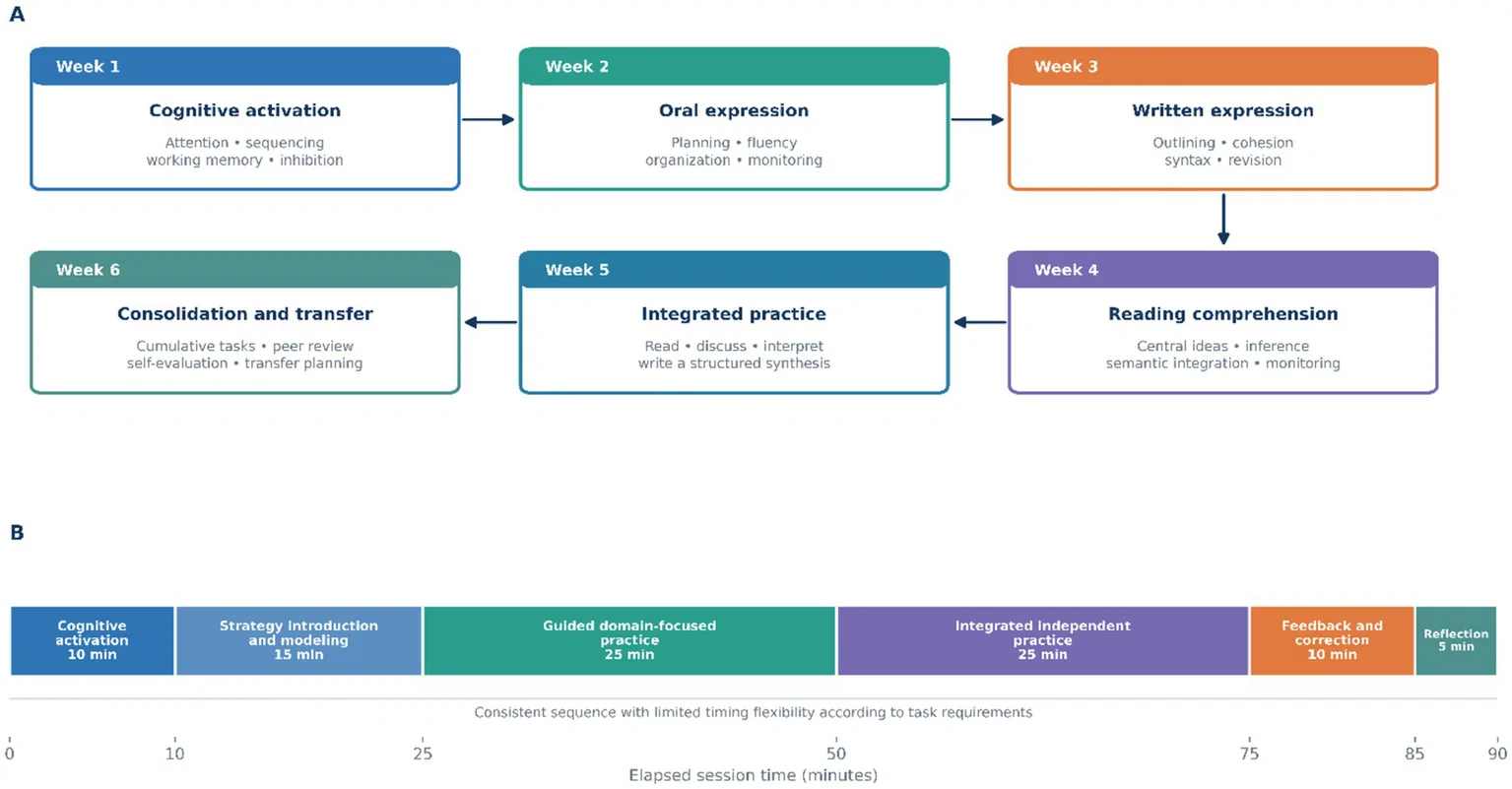
Structure of the 6-week cognitive-linguistic intervention. **(A)** Instructional progression across 6 weeks. **(B)** Standard structure of each 90-min session.

Oral activities included short academic presentations, oral summaries, narrative sequencing, concept explanation, and evidence-based arguments. Written activities required planning, production, and revision of 250–300-word analytical responses. Reading activities used academic texts followed by questions assessing explicit information, inference, semantic integration, and comprehension monitoring. Integrated activities required participants to read a text, discuss its central arguments, and produce an oral or written synthesis (Figure 2).

### Intervention delivery and fidelity

The intervention was delivered using a common manual specifying session objectives, activity sequence, materials, approximate duration, and feedback procedures. Before implementation, instructors completed 6 h of orientation covering intervention delivery, time management, attendance documentation, feedback, and fidelity assessment.

A six-item checklist was completed after each session to document whether the instructor stated the objectives, delivered the cognitive-activation activity, completed the domain-focused and integrated activities, provided structured feedback, and conducted metacognitive reflection. Each component was coded as delivered or not delivered as planned. Fidelity was calculated as:


$$\begin{array}{l} \text{Implementation fidelity}\;(\%)=\text{Number of components}\;\\ \text{delivered}\;\mathrm{as}\;\text{planned}\backslash \text{Number of planned components}\times 100. \end{array}$$

Attendance was recorded separately. Participants were required to attend at least 15 sessions, equivalent to 83.3% of the program, for inclusion in the paired analysis.

### Cognitive-linguistic assessment

Cognitive-linguistic performance was measured using a researcher-developed battery assessing oral expression, written expression, and reading comprehension. Each domain was scored from 0 to 20, and the three domain scores were summed to produce a global score ranging from 0 to 60. Higher values indicated stronger academic language performance. The instrument assessed structured academic performance and was not intended as a clinical or neuropsychological diagnostic measure. The assessment structure is summarized in Table 1, and example prompts and questions are provided in Supplementary Table S2.

**Table 1:** Structure and scoring of the cognitive-linguistic assessment.

Domain	Assessment task	Scoring criteria	Score
Oral expression	Three-minute response following 5 min of preparation	Fluency and lexical access; organization; coherence and relevance; monitoring and correction	0–20
Written expression	A 250–300-word analytical response completed within 30 min	Content development; organization and cohesion; syntactic accuracy; revision and clarity	0–20
Reading comprehension	Academic text followed by structured questions	Explicit information; inference; semantic integration; comprehension monitoring and critical interpretation	0–20
Global score	Sum of the three domain scores	Overall performance across the three domains	0–60

Each domain comprised four criteria scored from 0 to 5. The global score was calculated as oral expression + written expression + reading comprehension.

### Assessment administration and scoring

Oral expressions were assessed individually. Participants received an academic prompt, prepared for 5 min, and delivered an approximately 3-min response that was audio-recorded. Written expressions and reading comprehension were administered under standardized classroom conditions. Participants were given 30 min to complete each task.

Parallel assessment forms were used at baseline and post-intervention. Prompt and reading passages differed between occasions but were matched for format, approximate length, linguistic complexity, topic familiarity, scoring criteria, administration time, and maximum score.

Oral and written responses were independently evaluated by two trained assessors using analytic rubric. Each criterion was scored from 0, indicating no relevant or assessable performance, to 5, indicating complete, precise, coherent, and well-monitored performance. Assessors completed a calibration exercise before formal scoring and were blinded to participant identity and assessment occasion. Their mean rating was retained as the final score. Reading-comprehension responses were scored using a prespecified answer key. The complete analytic scoring rubric and performance descriptors are provided in Supplementary Table S3.

### Instrument development and measurement quality

The assessment battery and rubric were reviewed by five specialists: two in language education and one each in educational psychology, cognitive-linguistic assessment, and research methodology. Experts evaluated the relevance, clarity, coherence, and representativeness of the tasks using a four-point scale. The revised instrument was piloted with 20 undergraduate students who did not participate in the main study. Pilot testing evaluated instructional clarity, task duration, difficulty, and scoring feasibility.

Content validity was assessed using Aiken’s *V*. Internal consistency was evaluated using Cronbach’s alpha. Inter-rater agreement for oral and written responses was estimated using a two-way random-effects, absolute-agreement intraclass correlation coefficient with 95% confidence intervals. The instrument-development, expert-review, pilot-testing, and finalization procedures are summarized in Supplementary Table S4.

### Performance classification

For descriptive analysis, global scores were classified using prespecified educational thresholds: low performance, 0–35 points; moderate performance, 36–47 points; and high performance, 48–60 points. These categories corresponded to less than 60%, 60.0–79.9%, and at least 80% of the maximum score, respectively. They were used to summarize educational performance and were not interpreted as clinical categories of cognitive impairment or executive dysfunction.

### Procedure

After providing informed consent, participants completed the baseline assessment under standardized conditions. They then participated in the 6-week instructional program. The post-intervention assessment was administered immediately after the final intervention session using the parallel assessment form and the same administration and scoring procedures.

Responses were labeled with participant codes to maintain confidentiality and match observations across occasions. Before analysis, records were checked for transcription errors, duplicate entries, missing values, and scores outside their permissible ranges. No missing post-intervention values were imputed.

### Statistical analysis

Continuous outcomes were summarized as means and standard deviations. Individual change scores were calculated as post-intervention minus baseline values and reported as mean differences with 95% confidence intervals. Percentage change was calculated relative to the baseline mean.

Normality of the paired differences was evaluated using Q–Q plots and the Shapiro–Wilk test. Approximately normally distributed differences were analyzed using two-sided paired-samples *t*-tests. Wilcoxon signed-rank tests were used as sensitivity analyses when substantial departures from normality were identified. Holm-adjusted *p*-values were calculated for the global score and three domain comparisons.

Within-participant effect sizes were expressed as Cohen’s *d*_z_, calculated as the mean paired difference divided by the standard deviation of the individual differences. Effect sizes were reported with 95% confidence intervals and interpreted descriptively using 0.20, 0.50, and 0.80 as general reference values for small, moderate, and large effects.

Performance-level transitions were summarized in a 3 × 3 paired transition matrix and evaluated using the Stuart–Maxwell test of marginal homogeneity with an exact permutation procedure. Pearson correlations were calculated among oral-, written-, and reading-change scores to examine whether the magnitude of improvement was coordinated across domains. Participants were also classified as showing positive change in all three domains, a mixed response, or no positive domain change.

The robustness of the global standardized change was examined using leave-one-participant-out sensitivity analysis. Cohen’s *d*_z_ was recalculated after excluding each participant. All tests were two-sided with *α* = 0.05. Because the study used a one-group pre–post design, all analyses were considered exploratory and were interpreted as within-participant changes rather than definitive causal effects.

## Results

### Intervention implementation and measurement quality

**Figure 3:**
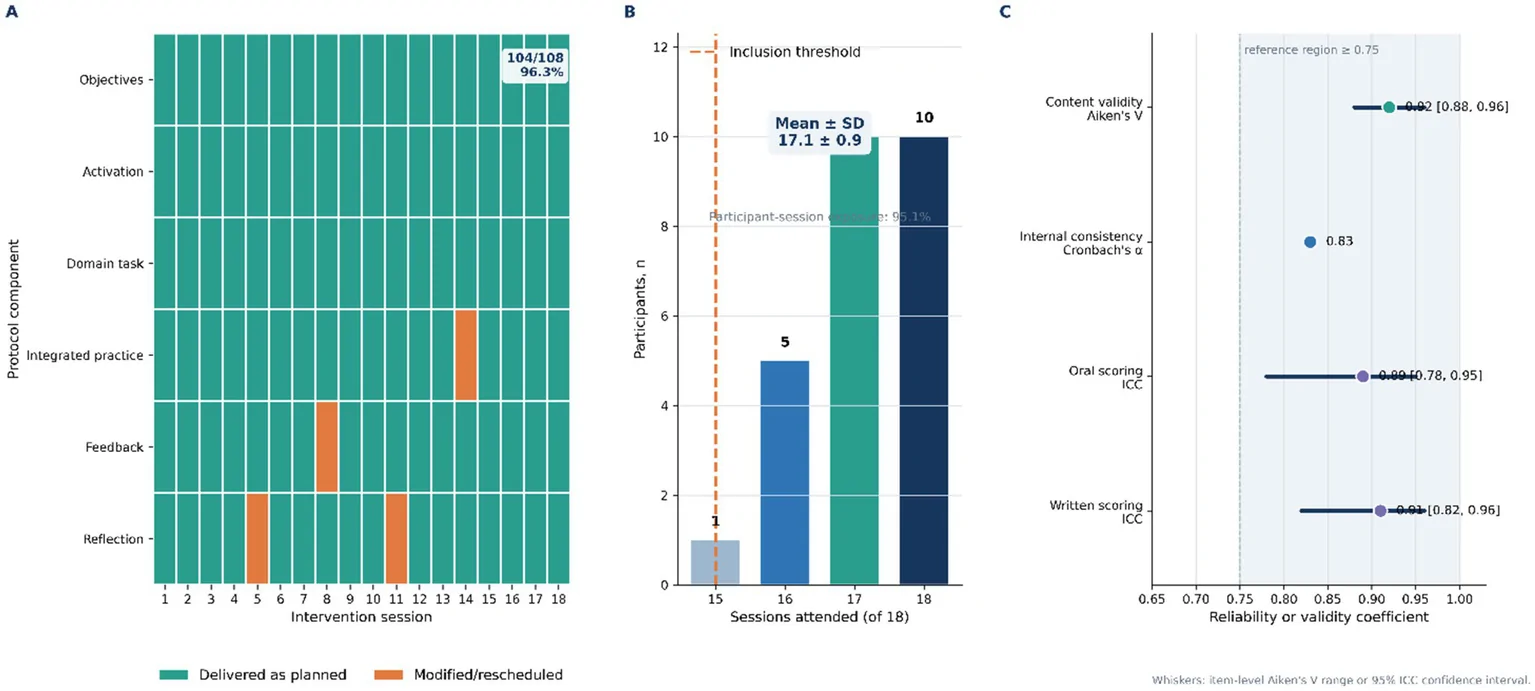
Intervention implementation, participant exposure, and measurement quality. Whiskers represent the item-level Aiken’s *V* range or 95% confidence intervals for the intraclass correlation coefficients. ICC, intraclass correlation coefficient; SD, standard deviation. **(A)** Session-level implementation fidelity. **(B)** Intervention attendance dose. **(C)** Measurement-quality evidence.

Implementation and measurement-quality indicators are summarized in Figure 3. Of the 108 planned intervention components, 104 were delivered as planned, corresponding to an overall fidelity of 96.3%. The four modified components consisted of two shortened metacognitive-reflection activities, one feedback activity delivered by the instructor rather than peers, and one integrated written task completed during the following session.

Attendance among the 26 analyzed participants ranged from 15 to 18 sessions, with a mean of 17.1 ± 0.9 sessions. Ten participants attended all 18 sessions, 10 attended 17 sessions, five attended 16 sessions, and one attended 15 sessions. Overall, participants completed 445 of 468 possible participant-session exposures, corresponding to 95.1% attendance exposure.

The assessment battery demonstrated acceptable measurement quality. Overall content validity was Aiken’s *V* = 0.92, with item-level values ranging from 0.88 to 0.96, while internal consistency was Cronbach’s *α* = 0.83. Inter-rater agreement was high for oral expression, ICC = 0.89, 95% CI [0.78, 0.95], and written expression, ICC = 0.91, 95% CI [0.82, 0.96]. These findings summarize intervention implementation, participant exposure, and measurement quality (Figure 3). Aggregate content-validity, internal-consistency, and inter-rater agreement estimates are reported in Supplementary Table S5.

### Changes in cognitive-linguistic performance

Post-intervention scores were higher than baseline scores for the global measure and all three domains (Table 2). The global cognitive-linguistic score increased by 11.53 points, from 45.35 ± 4.38 to 56.88 ± 5.54, corresponding to a 25.4% increase. The paired difference was statistically significant, *t*(25) = 12.54, Holm-adjusted *p* < 0.001, with a large standardized mean change (*d*_z_ = 2.46). Oral expression increased by 4.19 points (27.9%), written expression by 3.72 points (25.4%), and reading comprehension by 3.62 points (23.1%). All three paired comparisons remained statistically significant after Holm adjustment. The 95% confidence intervals excluded zero, indicating that the direction of the observed mean change was consistently positive across the assessed outcomes.

**Table 2:** Baseline and post-intervention cognitive-linguistic performance.

Outcome	Baseline mean ± SD	Post-intervention means ± SD	Mean change (95% CI)	Change (%)	Paired *t*(25)	Holm-adjusted *p*	Cohen’s *d*_z_
Global cognitive-linguistic score	45.35 ± 4.38	56.88 ± 5.54	11.53 (9.64–13.42)	25.4	12.54	<0.001	2.46 (1.67–3.23)
Oral expression	15.02 ± 2.11	19.21 ± 2.38	4.19 (3.26–5.12)	27.9	9.28	<0.001	1.82 (1.18–2.44)
Written expression	14.63 ± 2.27	18.35 ± 2.61	3.72 (2.79–4.65)	25.4	8.21	<0.001	1.61 (1.02–2.19)
Reading comprehension	15.70 ± 2.41	19.32 ± 2.46	3.62 (2.65–4.59)	23.1	7.65	<0.001	1.50 (0.93–2.06)

### Changes in performance-level classification

The distribution of global cognitive-linguistic performance shifted toward higher categories at post-intervention (Table 3). At baseline, 2 participants (7.7%) were classified at the low level, 22 (84.6%) at the moderate level, and 2 (7.7%) at the high level. At post-intervention, no participant remained in the low category, 5 participants (19.2%) were classified at the moderate level, and 21 (80.8%) were classified at the high level. Participant-level transitions showed that 21 students (80.8%) moved to a higher category, whereas 5 (19.2%) remained in the same category. No participant moved to a lower category. The paired marginal distributions differed significantly, Stuart–Maxwell *χ*^2^(2) = 21.00, exact *p* < 0.001.

**Table 3:** Participant-level transitions in global cognitive-linguistic performance.

Baseline performance level	Post: low, *n*	Post: moderate, *n*	Post: high, *n*	Baseline total, *n* (%)
Low	0	2	0	2 (7.7)
Moderate	0	3	19	22 (84.6)
High	0	0	2	2 (7.7)
Post-intervention total	0 (0.0)	5 (19.2)	21 (80.8)	26 (100.0)

Performance was classified using the prespecified global-score thresholds: low, 0–35; moderate, 36–47; and high, 48–60. Percentages were calculated using the 26 paired observations.

### Multidimensional analysis of cognitive-linguistic change following the intervention

**Figure 4:**
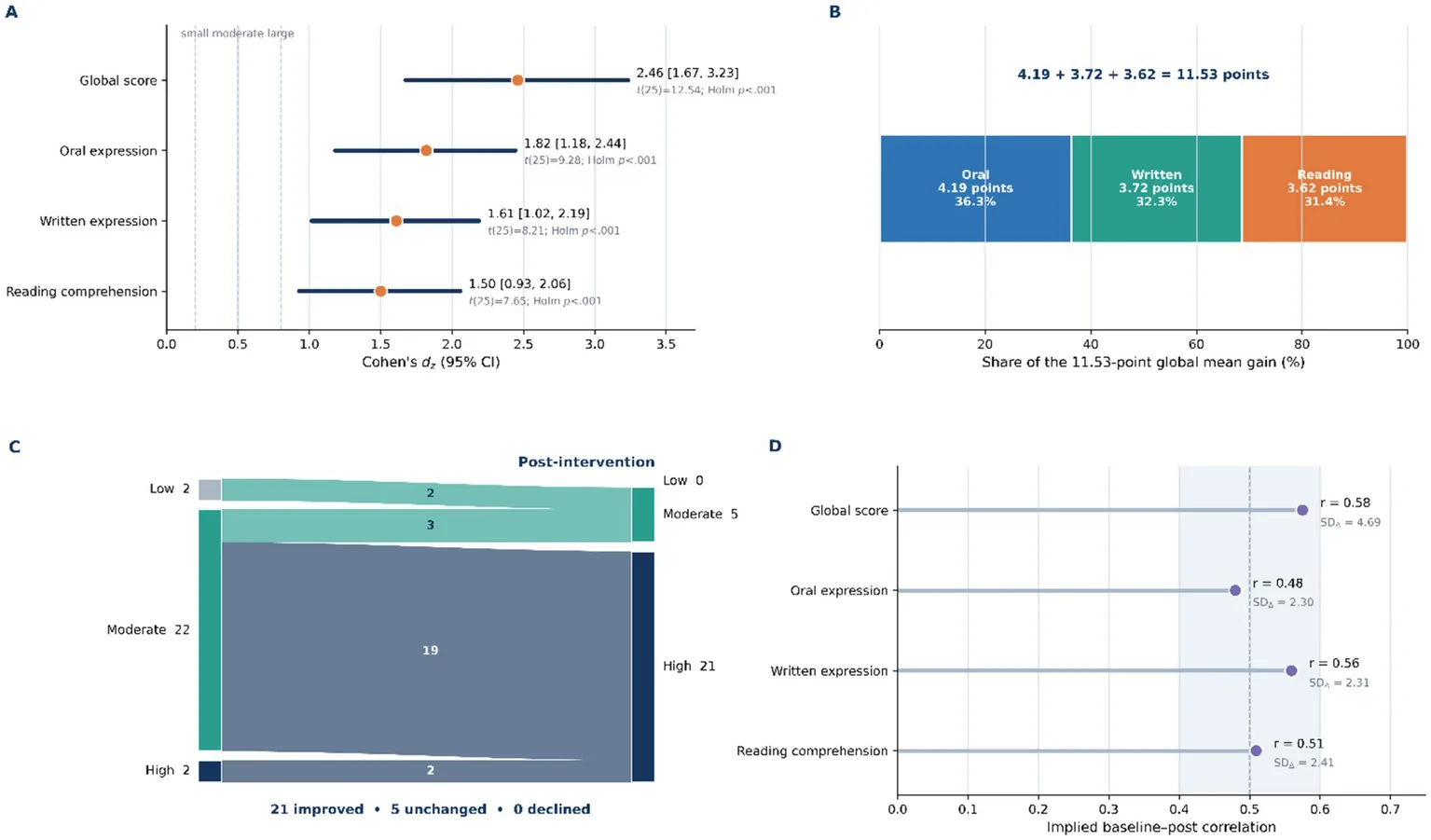
Multidimensional summary of cognitive-linguistic change following the intervention. **(A)** Magnitude, precision, and adjusted inference. **(B)** Additive decomposition of the global gain. **(C)** Participant-level category flow. **(D)** Paired-score dependence implied by reported statistics.

Figure 4 summarizes complementary aspects of the observed cognitive-linguistic changes. Panel A shows large standardized within-participant increases for the global score (*d*_z_ = 2.46, 95% CI [1.67, 3.23]), oral expression (*d*_z_ = 1.82, 95% CI [1.18, 2.44]), written expression (*d*_z_ = 1.61, 95% CI [1.02, 2.19]), and reading comprehension (*d*_z_ = 1.50, 95% CI [0.93, 2.06]). All confidence intervals remained above the conventional large-effect reference value of 0.80, and all comparisons remained statistically significant after Holm adjustment (*p* < 0.001). These estimates indicate substantial pre–post differences within the participating group, although they do not establish that the intervention alone caused the improvement.

Panel B demonstrates that the 11.53-point increase in the global score was additively distributed across oral expression (4.19 points; 36.3%), written expression (3.72 points; 32.3%), and reading comprehension (3.62 points; 31.4%). Thus, the global increase was not dominated by a single domain. These percentages represent arithmetic contributions to the summed global score, not proportions of explained variance. Panel C shows that 21 participants (80.8%) moved to a higher performance category, five (19.2%) remained in the same category, and none moved to a lower category. Specifically, two participants moved from low to moderate performance, 19 moved from moderate to high performance, three remained moderate, and two remained high.

Panel D describes the dependence structure required by the reported repeated measures statistics. The implied baseline–post-intervention correlations ranged from 0.48 to 0.58, indicating moderate correspondence between participants’ relative performance at the two assessment occasions. The change-score standard deviations ranged from 2.30 to 2.41 points for the three individual domains and were 4.69 points for the global score. These quantities are derived from the reported means, standard deviations, and *d*_z_ estimates rather than independently fitted outcomes. Collectively, the panels show large, broadly distributed within-participant improvements accompanied by a pronounced upward movement in performance classification; however, the absence of a control group requires cautious interpretation regarding causality, transfer, and durability.

### Individual-level robustness and cross-domain consistency

**Figure 5:**
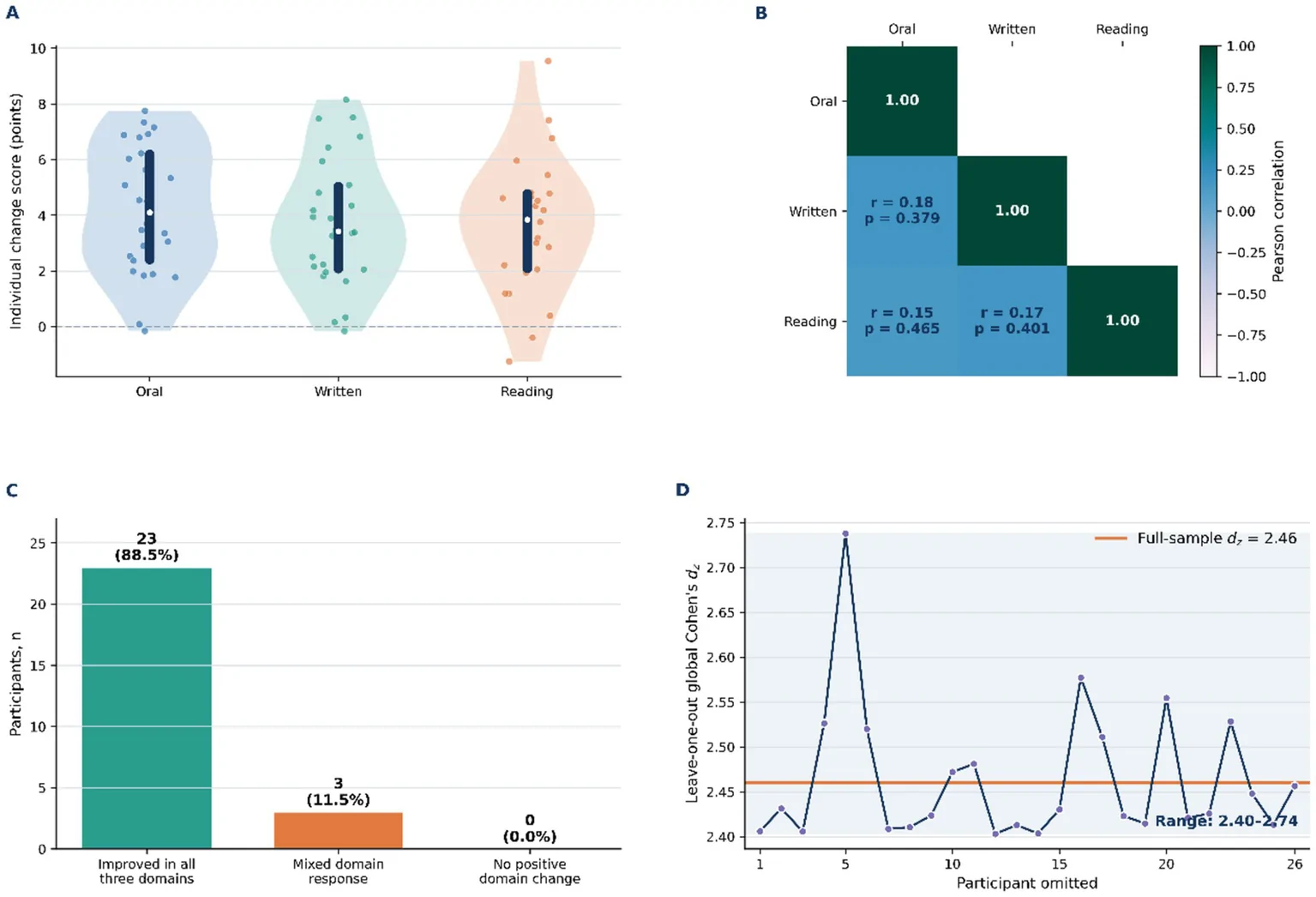
Individual-level robustness and cross-domain consistency of cognitive-linguistic change. Each point represents Cohen’s *d*_z_ after sequential omission of one participant, and the horizontal line indicates the full-sample estimate. Change-score distributions, domain coordination, response profiles, and influence diagnostics (*n* = 26). **(A)** Distribution and heterogeneity of individual change. **(B)** Coordination among domain-specific changes. **(C)** Multidomain response profiles. **(D)** Leave-one-participant-out sensitivity.

The individual change-score distributions showed considerable variation in the magnitude of improvement within each domain (Figure 5). Most observations were above zero, although isolated non-positive changes were present in each distribution. Twenty-three participants (88.5%) showed positive changes in oral expression, written expression, and reading comprehension simultaneously, whereas three participants (11.5%) exhibited a mixed response across domains. No participant showed non-positive change across all three domains. Thus, the predominant response profile was multidomain improvement rather than improvement restricted to only one assessed area. Correlations among domain-specific changes were positive but weak and statistically non-significant. Oral- and written-expression changes were correlated at *r* = 0.18 (*p* = 0.379), oral-expression and reading-comprehension changes at *r* = 0.15 (*p* = 0.465) and written-expression and reading-comprehension changes at *r* = 0.17 (*p* = 0.401). These results indicate that participants who showed comparatively large improvement in one domain did not necessarily exhibit equally large improvement in another. Therefore, the findings support a common positive direction of change across domains at the group level, but they do not demonstrate strong individual-level coupling among the magnitudes of domain-specific gains.

The leave-one-participant-out analysis indicated that the global standardized change remained large regardless of which observation was omitted. Cohen’s *d*_z_ estimates ranged from 2.40 to 2.74, compared with the full-sample estimate of 2.46. No single participant reduced the standardized change below the conventional large-effect reference value. This sensitivity analysis suggests that the global result was not determined exclusively by one influential observation, although the small sample and absence of a comparison group continue to limit causal interpretation.

## Discussion

The present study examined changes in university students’ cognitive-linguistic performance following a 6-week structured instructional program. Higher post-intervention performance was observed for global measures and for oral expression, written expression, and reading comprehension. The consistency of the direction of change across these outcomes suggests that improvement was not confined to a single communicative skill. Nevertheless, because the study employed a one-group pre–post design, the findings represent changes associated with participation in the program rather than definitive evidence that the intervention alone produced those changes.

The findings are compatible with theoretical perspectives that describe executive control and language processing as related components of complex academic performance. Executive functions support the maintenance of task goals, inhibition of irrelevant information, flexible adjustment of strategies, and monitoring of performance (Diamond, 2013; Miyake and Friedman, 2012). These processes are relevant to oral communication, writing, and reading because each domain requires students to select information, organize it coherently, and evaluate whether their response satisfies the task requirements. The improvement observed across the three domains may therefore reflect repeated practice in applying regulatory strategies within structured language activities.

### Oral expression

The change in oral-expression performance may be related to the repeated use of planning, sequencing, explanation, argumentation, and self-monitoring activities. Effective oral production requires speakers to maintain a communicative objective, retrieve appropriate vocabulary, organize propositions, suppress irrelevant content, and monitor speech while it is being produced (Levelt, 1999). These demands involve interactions between language processing and executive regulation rather than isolated verbal fluency.

The intervention provided opportunities to prepare short responses, summarize information, explain academic concepts, and construct evidence-based arguments. Instructor and peer feedback may also have helped participants recognize weaknesses in organization, relevance, and clarity. This interpretation is consistent with the position that sustained engagement in cognitively demanding tasks can support executive control when the activities require active monitoring and progressively challenging performance (Diamond and Ling, 2016). However, the present study did not administer an independent executive-function test; therefore, improved oral performance should not be interpreted as direct evidence of generalized executive enhancement.

### Written expression

The improvement in written expression is consistent with cognitive models that regard writing as a coordinated process involving planning, formulation, transcription, evaluation, and revision. Kellogg (2008) emphasized that these processes compete for limited working-memory resources and become more efficient through structured practice (Kormos, 2006). In the present program, participants prepared outlines, developed academic responses, organized supporting information, and revised texts for syntax, cohesion, coherence, and communicative clarity. Such activities may have helped students distribute their cognitive resources more effectively across the different stages of writing.

The result is also relevant to the academic challenges encountered by university students. Wette and Furneaux (2018) described the difficulties students may experience when adapting to the linguistic, organizational, and rhetorical expectations of academic discourse. Explicit instruction in planning, textual organization, and revision can make these expectations more visible and manageable (De La Paz and Graham, 2002). The present findings therefore support the pedagogical value of treating writing as a recursive and strategically regulated activity rather than evaluating only the final written product.

Nevertheless, the written-assessment task was closely aligned with the activities practiced during the intervention. Improvement may consequently represent strengthened performance on similar academic writing tasks rather than transfer to all forms of written communication. Future studies should assess transfer using genres, topics, and task conditions not included in the training program.

### Reading comprehension

The reading-comprehension findings can be interpreted through models emphasizing the integration of lexical, semantic, and contextual information. Perfetti and Stafura (2014) proposed that successful comprehension depends on the quality of word knowledge and the reader’s ability to integrate information into a coherent representation. Comprehension also requires the generation of inferences and monitoring of inconsistencies or incomplete interpretations.

The intervention incorporated identification of central ideas, recognition of supporting information, inference generation, semantic integration, and comprehension-monitoring questions. These activities may have encouraged participants to move beyond literal retrieval and engage more actively with relationships among ideas. The use of discussion and written synthesis may have further required students to reorganize textual information and express their interpretation in another format.

These results should, however, be limited to the types of academic texts and questions used in the study. The findings do not establish improvement in reading across different disciplines, text genres, or levels of complexity. Parallel or unpracticed assessments would be needed to determine whether the observed change extends beyond the materials and strategies used during the intervention.

### Multidomain change and cognitive-linguistic integration

The positive direction of change across oral expression, written expression, and reading comprehension is broadly consistent with an integrated view of cognitive-linguistic performance. These domains involve distinguishable outputs, but they share demands related to attention, working memory, semantic processing, planning, and monitoring. Miyake and Friedman (2012) similarly argued that executive functioning contains both shared and specific components. Improvement across domains can therefore occur through common regulatory processes while retaining domain-specific characteristics.

The individual-level analysis provided an important qualification for this interpretation. Although most participants demonstrated positive changes across all three domains, correlations among the magnitudes of domain-specific changes were weak (Olaru et al., 2023). Participants who showed relatively large improvement in one domain did not necessarily show improvement of the same magnitude in another. This pattern suggests that a common positive direction at the group level does not imply that the underlying changes were governed by one unitary mechanism (Martín-Gascón, 2024; Snowling and Hulme, 2021; Wilson and Buttrick, 2016).

Differences among participants may have reflected baseline skill profiles, engagement with activities, prior experience, or variation in the strategies used during the program (Nielsen et al., 2009). Oral, written, and reading tasks may also have responded differently to feedback and practice. Accordingly, the findings support multidomain improvement but provide limited evidence for tightly coupled individual changes. Claims of complete cognitive integration or global executive-linguistic reorganization would therefore be too strong.

### Comparison with previous intervention research

The present findings are broadly consistent with Rogowsky et al. (2013), who reported improvements in reading and writing following cognitive and linguistic skills training among college students. Both studies indicate that structured practice combining cognitive and language demands may support academic communication skills. The results also agree with the meta-analysis by de Boer et al. (2014), which showed that the effectiveness of educational interventions can depend on characteristics such as instructional organization, duration, implementation, and alignment with intended outcomes.

The intervention’s structured progression may be particularly relevant. It began with cognitive activation, moved through oral, written, and reading activities, and concluded with integrated practice and metacognitive reflection. Liu and Qin (2024) similarly emphasized the potential value of cognitively informed language pedagogies while also noting variation in intervention approaches and outcomes. The current program’s combination of explicit modeling, guided practice, independent application, feedback, and reflection may have provided a coherent instructional sequence for applying cognitive-linguistic strategies.

Metacognitive reflection may also have contributed to the observed pattern. Kuiper (2002) argued that reflective use of self-regulated learning strategies can strengthen awareness of how learners’ approach and monitor tasks. Asking participants to identify the strategies used, evaluate their effectiveness, and consider their application in other academic contexts may have encouraged more deliberate regulation of learning. However, metacognitive change was not measured independently in this study, so its contribution remains a plausible instructional explanation rather than a tested mechanism.

### Contradictory evidence and transfer limitations

The findings must also be considered alongside research questioning broad transfer from cognitive training. Melby-Lervåg et al. (2016) concluded that working-memory training generally improves trained or closely related tasks but provides limited evidence of far transfer to intelligence or other untrained abilities. Diamond and Ling (2016) likewise cautioned that apparent executive-function improvements may depend on task similarity, implementation characteristics, participant expectations, and study design.

These concerns are directly relevant because the instructional and assessment activities in the present study were conceptually aligned. Participants practiced oral organization, written production, reading comprehension, and metacognitive monitoring and were assessed using tasks involving similar competencies. Such alignment is pedagogically appropriate, but it makes it difficult to distinguish improvement in the targeted academic skills from broader cognitive transfer.

The findings should therefore be interpreted as evidence of near-task cognitive-linguistic improvement. They do not establish enhancement of general intelligence, broad executive functioning, neural plasticity, or cognitive resilience. Demonstration of broader transfer would require validated independent measures and assessment tasks that differ substantially from those practiced during the intervention.

### Implementation and measurement quality

High attendance and general consistent implementation strengthen confidence that the participating students received the planned instructional exposure. The fidelity assessment showed that most intervention components were delivered according to the standardized session structure, with only limited modifications. This reduces concern that the findings resulted from highly inconsistent implementation.

The assessment battery also demonstrated acceptable content validity, internal consistency, and inter-rater agreement. Expert review and pilot testing supported the relevance and clarity of the tasks, while independent scoring improved the consistency of oral- and written-expression evaluation. These measurement indicators strengthen the reproducibility of the assessment procedure within the present sample.

Implementation of fidelity and measurement reliability do not, however, establish causal validity. A consistently delivered intervention may still coincide with changes caused partly by repeated testing, concurrent coursework, or expectancy effects. Similarly, a reliable instrument can measure change consistently without demonstrating that the intervention was its sole cause.

### Pedagogical implications

The results support the use of instructional sequences that combine language performance with explicit cognitive and metacognitive strategies. University instructors may integrate short planning exercises, oral explanation, guided reading, written synthesis, feedback, and reflection within the same learning cycle. Such an approach can help students recognize that reading, speaking, and writing are connected academic practices rather than isolated classroom activities.

For oral activities, students may benefit from brief planning time, explicit organizational frameworks, and criteria-based feedback. Written activities can incorporate outlines, staged drafting, peer review, and revision checklists. Reading instruction can emphasize inference generation, connection of information across paragraphs, and monitoring of understanding. Short reflective questions may then help students identify which strategies were effective and how they could be transferred to other academic tasks.

These recommendations should be implemented as instructional practices rather than clinical cognitive training. The current findings do not justify identifying students as cognitively impaired or claiming that the program prevents cognitive decline. Its practical contribution lies in organizing university language instruction around planning, integration, monitoring, and reflection.

### Limitations and future research

Several limitations should be acknowledged. First, the absence of a control group prevents clear separation of intervention-associated change from testing effects, maturation, concurrent coursework, expectancy effects, and regression to the mean. A future study should include an active comparison group receiving equivalent instructional time and attention. The sample was small and drawn from an academic program at a single university. This limits precision and generalizability to students from other disciplines, institutions, age groups, or linguistic backgrounds. Larger multisite samples would allow examination of whether intervention responses differ according to baseline performance, academic year, attendance, or participant characteristics. The post-intervention assessment was administered immediately after the program. The study therefore cannot determine whether the observed changes were retained. Alignment between the intervention and assessment may have increased performance on closely related tasks. Future research should use validated external instruments, parallel forms, and untrained transfer tasks. Assessors should remain blinded to participant identity and assessment occasion. Study evaluated behavioral academic performance and did not include neuropsychological, neurophysiological, or neurological measures. Consequently, the findings do not demonstrate neural reorganization, stable cognitive attractor states, generalized executive-function enhancement, or protection against cognitive decline.

## Conclusion

This study found substantial improvements in global cognitive-linguistic performance and in oral expression, written expression, and reading comprehension following a 6-week structured didactic intervention. The multidomain pattern of change suggests that integrating executive-control demands, language processing, semantic organization, guided practice, feedback, and reflection may support several related academic language abilities within a single instructional framework. High attendance and implementation fidelity indicate that the intervention was feasible for the participating undergraduate students.

These findings should nevertheless be interpreted as preliminary. The small sample, recruitment from one academic program, absence of a control group, and lack of long-term follow-up prevent firm causal conclusions and limit generalizability. Improvements cannot be separated conclusively from maturation, repeated testing, concurrent instruction, or other uncontrolled influences.

Pedagogically, the results support incorporating structured cognitive-linguistic activities into communication and literature courses. Instructors may use tasks that require students to plan and organize ideas, connect semantic information, monitor comprehension, revise oral and written responses, and apply feedback across progressively demanding activities. Future randomized or controlled studies with larger and more diverse samples, blinded assessment, preregistered analyses, and delayed follow-up measurements are required to determine the intervention’s comparative effectiveness, durability, and applicability across educational settings.

## Data Availability

The raw data supporting the conclusions of this article will be made available by the authors, without undue reservation.
