# Supplementary material for: Changes in university students cognitive-linguistic performance following a structured didactic intervention
Source: Front Psychol. 2026 Sep 3;17:1779389. doi: 10.3389/fpsyg.2026.1779389 (PMC13581959; doi:10.3389/fpsyg.2026.1779389)
Supplement: Supplementary file 1 [file Table_1.DOCX]

**Supplementary materials**

Table S1. Complete example of a 90-minute cognitive-linguistic intervention session

| **Session element** | **Description** |
| --- | --- |
| Session focus | Semantic integration and written synthesis |
| Learning objective | Identify relationships among ideas in an academic text and produce a coherent, evidence-based synthesis. |
| Targeted processes | Sustained attention, working-memory updating, identification of central ideas, inference generation, semantic integration, planning, monitoring, and revision. |
| Materials | Two academic passages, semantic-organization worksheet, analytical-writing sheet, criteria-based feedback checklist, and metacognitive-reflection form. |
| Delivery format | Instructor modeling, guided practice, independent application, structured feedback, and individual reflection. |

| **Stage** | **Time** | **Activity and procedure** | **Expected evidence** |
| --- | --- | --- | --- |
| Cognitive activation | 10 min | Students organize six concept statements into a logical sequence and briefly explain the relationship between adjacent statements. | Completed sequence and oral justification. |
| Strategy introduction and modeling | 15 min | The instructor models how to identify a central claim, supporting evidence, causal relationships, contrasts, and implicit connections in a short academic passage. A think-aloud procedure demonstrates how comprehension is monitored. | Modeled semantic organizer. |
| Guided domain-focused practice | 25 min | Students read a second passage and complete a structured organizer identifying the central idea, supporting information, semantic relationships, and one justified inference. Responses are discussed with instructor guidance. | Completed organizer and corrected inference. |
| Integrated independent practice | 25 min | Students prepare a 250–300-word synthesis integrating the central claim, supporting evidence, and one inferred implication without copying sentences directly from the source. | Individual analytical synthesis. |
| Feedback and correction | 10 min | The response is reviewed using criteria for relevance, organization, semantic integration, cohesion, and clarity. Each student identifies and revises at least one weakness. | Feedback checklist and revised passage. |
| Metacognitive reflection | 5 min | Students identify the strategy used, evaluate its usefulness, describe the most difficult part of the task, and state one academic context in which the strategy could be applied again. | Completed reflection form. |

Table S2. Examples of cognitive-linguistic assessment tasks

| **Domain** | **Example task** | **Administration** | **Scoring focus** | **Score** |
| --- | --- | --- | --- | --- |
| Oral expression | After five minutes of preparation, explain and defend a position on the following academic issue: ‘Should university courses combine independent learning with structured collaborative activities?’ Organize the approximately three-minute response using a clear introduction, supporting arguments, and conclusion. | Individual administration; response audio-recorded. | Fluency and lexical access; organization; coherence and relevance; monitoring and correction. | 0–20 |
| Written expression | Write a 250–300-word analytical response explaining how critical reading contributes to effective academic writing. Support the position with logically organized reasons and examples and revise the response before submission. | Standardized classroom conditions; 30 minutes. | Content development; organization and cohesion; syntactic accuracy; revision and clarity. | 0–20 |
| Reading comprehension | After reading the supplied academic passage: (a) identify its central claim; (b) locate two supporting details; (c) explain one implied relationship between ideas; (d) interpret a key term from context; and (e) evaluate whether the evidence supports the conclusion. | Standardized classroom conditions; 30 minutes; prespecified answer key. | Explicit information; inference; semantic integration; comprehension monitoring and critical interpretation. | 0–20 |

| **Matching characteristic** | **Control applied** |
| --- | --- |
| Task format | Equivalent response format and number of required components. |
| Text and prompt length | Comparable approximate length across assessment occasions. |
| Linguistic complexity | Comparable vocabulary, sentence complexity, and inferential demand. |
| Topic familiarity | Topics selected to minimize advantage from specialized prior knowledge. |
| Administration | Equivalent instructions, preparation time, completion time, and testing setting. |
| Scoring | Identical analytic criteria, score ranges, answer-key principles, and assessor procedures. |

**Table S3. Analytic scoring rubric for oral expression, written expression, and reading comprehension**

| **Criterion** | **Score** | **Performance descriptor** |
| --- | --- | --- |
| Fluency and lexical access | 0 | No relevant or assessable oral response. |
|  | 1 | Produces isolated words or fragments; frequent breakdowns prevent communication. |
|  | 2 | Produces a limited response with long pauses, repetition, and restricted vocabulary. |
|  | 3 | Maintains a generally understandable response despite noticeable hesitation or lexical limitations. |
|  | 4 | Speaks fluently with appropriate academic vocabulary and only minor hesitation. |
|  | 5 | Speaks consistently and precisely, using varied academic vocabulary with effortless lexical access. |
| Organization | 0 | No identifiable organization. |
|  | 1 | Ideas are disconnected and no recognizable sequence is established. |
|  | 2 | A limited sequence is present, but the response lacks a clear opening, development, or conclusion. |
|  | 3 | The response has an identifiable structure, although some transitions or relationships are unclear. |
|  | 4 | Ideas are logically sequenced with a clear opening, development, transitions, and conclusion. |
|  | 5 | The response is strategically structured, with seamless progression and effective emphasis of key ideas. |
| Coherence and relevance | 0 | No relevant content is provided. |
|  | 1 | Content is largely unrelated to the prompt or internally contradictory. |
|  | 2 | Some relevant ideas are provided, but connections are weak or important information is omitted. |
|  | 3 | Most content is relevant and understandable, with occasional weak or unsupported connections. |
|  | 4 | Content remains relevant and coherent, and claims are supported with appropriate explanations or examples. |
|  | 5 | Content is consistently focused, logically integrated, and supported by precise and persuasive evidence. |
| Monitoring and correction | 0 | No evidence of monitoring or correction. |
|  | 1 | Does not recognize errors that substantially obstruct meaning. |
|  | 2 | Recognizes or corrects occasional errors but monitoring is inconsistent. |
|  | 3 | Corrects several errors and generally maintains the intended meaning. |
|  | 4 | Monitors the response effectively and independently corrects most disruptions. |
|  | 5 | Continuously monitors clarity and accuracy and makes timely, unobtrusive corrections that strengthen the response. |

## Written expression

| **Criterion** | **Score** | **Performance descriptor** |
| --- | --- | --- |
| Content development | 0 | No relevant or assessable written response. |
|  | 1 | Provides isolated or largely irrelevant statements with no developed position. |
|  | 2 | Presents a limited position with minimal explanation or supporting information. |
|  | 3 | Develops a recognizable position with some relevant support, although elaboration is uneven. |
|  | 4 | Develops a clear position using relevant reasons, evidence, or examples. |
|  | 5 | Develops a precise and insightful position through well-selected, fully integrated evidence and explanation. |
| Organization and cohesion | 0 | No identifiable organization or cohesion. |
|  | 1 | Ideas are fragmented, and relationships among sentences are unclear. |
|  | 2 | Some organizational pattern is visible, but paragraphing and cohesive links are weak. |
|  | 3 | The response is generally organized, with occasional ineffective transitions or paragraph relationships. |
|  | 4 | The response has a logical structure, effective paragraphing, and appropriate cohesive devices. |
|  | 5 | The response is strategically organized, with seamless cohesion and a clear progression of ideas. |
| Syntactic accuracy | 0 | No assessable sentence-level production. |
|  | 1 | Persistent grammatical and syntactic errors substantially obstruct meaning. |
|  | 2 | Frequent errors limit clarity, although parts of the response remain understandable. |
|  | 3 | Generally comprehensible syntax with several errors that do not substantially obstruct meaning. |
|  | 4 | Mostly accurate and varied sentence structures with only minor errors. |
|  | 5 | Consistently accurate, controlled, and appropriately varied syntax that supports precise communication. |
| Revision and clarity | 0 | No evidence of revision or a communicatively interpretable response. |
|  | 1 | The response remains unclear and shows no meaningful correction. |
|  | 2 | Limited revision is evident, but avoidable ambiguity and errors remain. |
|  | 3 | Some effective revision improves clarity, although several weaknesses remain. |
|  | 4 | Revision improves precision, concision, and readability, with few remaining weaknesses. |
|  | 5 | Revision is comprehensive and purposeful, producing a precise, concise, and polished response. |

## Reading comprehension

| **Criterion** | **Score** | **Performance descriptor** |
| --- | --- | --- |
| Explicit information | 0 | No correct identification of explicitly stated information. |
|  | 1 | Identifies only isolated details and substantially misrepresents the central information. |
|  | 2 | Identifies some correct information but omits or confuses important details. |
|  | 3 | Identifies the main explicit information with minor omissions or inaccuracies. |
|  | 4 | Accurately identifies the central idea and relevant supporting information. |
|  | 5 | Precisely identifies, prioritizes, and relates all essential explicitly stated information. |
| Inference | 0 | No relevant inference is produced. |
|  | 1 | Produces an unsupported or textually contradictory inference. |
|  | 2 | Produces a partially plausible inference with weak textual support. |
|  | 3 | Produces a reasonable inference supported by some relevant textual evidence. |
|  | 4 | Produces a well-supported inference that integrates information from the text. |
|  | 5 | Produces a precise and insightful inference by integrating multiple textual cues and explaining the reasoning. |
| Semantic integration | 0 | No meaningful integration of textual information. |
|  | 1 | Treats ideas as disconnected or combines them inaccurately. |
|  | 2 | Recognizes limited relationships among ideas but constructs an incomplete representation. |
|  | 3 | Integrates the main ideas into a generally coherent representation with minor gaps. |
|  | 4 | Accurately connects meanings, relationships, and contextual information across the text. |
|  | 5 | Constructs a comprehensive and nuanced representation integrating meanings, context, and prior textual information. |
| Monitoring and critical interpretation | 0 | No evidence of comprehension monitoring or interpretation. |
|  | 1 | Does not recognize major inconsistencies or bases judgments on information absent from the text. |
|  | 2 | Shows limited monitoring and provides a weakly justified evaluation. |
|  | 3 | Recognizes some comprehension difficulty and provides a generally reasonable evaluation. |
|  | 4 | Monitors understanding, resolves inconsistencies, and evaluates claims using relevant evidence. |
|  | 5 | Actively monitors and revises interpretation and provides a precise, evidence-based critical evaluation. |

Table S4. Development, expert review, pilot testing, and finalization of the assessment battery

| **Stage** | **Purpose** | **Procedure** | **Documented output** |
| --- | --- | --- | --- |
| Construct specification | Define oral expression, written expression, and reading comprehension as related but distinct academic-language domains. | Domain definitions, task formats, scoring criteria, administration conditions, and score ranges were specified before the main study. | Three domain scores of 0–20 and a summed global score of 0–60. |
| Initial task and rubric development | Translate the construct specifications into assessable performance tasks. | Oral, written, and reading tasks and analytic scoring criteria were drafted to represent planning, organization, coherence, inference, semantic integration, monitoring, and revision. | Initial assessment battery and scoring rubric. |
| Expert content review | Evaluate content relevance and clarity. | Five specialists—two in language education and one each in educational psychology, cognitive-linguistic assessment, and research methodology—rated relevance, clarity, coherence, and representativeness using a four-point scale. | Expert ratings used for Aiken’s V and revision decisions. |
| Revision after expert review | Address weaknesses identified by the panel. | Task instructions, wording, criterion definitions, and correspondence between tasks and scoring criteria were reviewed and refined where indicated by expert judgments. | Revised battery prepared for pilot administration. |
| Pilot testing | Examine feasibility before the main study. | The revised battery was administered to 20 undergraduate students who did not participate in the main study. Instructional clarity, task duration, difficulty, administration feasibility, and scoring feasibility were examined. | Final adjustments to instructions and administration procedures. |
| Assessor calibration | Promote consistent application of the rubric. | Two assessors reviewed the rubric and completed a calibration exercise before independently scoring formal oral and written responses. | Common interpretation of scoring criteria before formal assessment. |
| Parallel-form matching | Reduce direct recall while maintaining comparability across occasions. | Baseline and post-intervention prompts and passages differed but were matched for format, approximate length, linguistic complexity, topic familiarity, administration time, scoring criteria, and maximum score. | Parallel baseline and post-intervention forms. |

Table S5. Measurement-quality evidence for the cognitive-linguistic assessment battery

| **Quality property** | **Statistic** | **Component** | **Estimate** | **Basis** |
| --- | --- | --- | --- | --- |
| Content validity | Aiken’s V | Overall assessment battery | 0.92 | Five experts; four-point ratings of relevance, clarity, coherence, and representativeness. |
| Content validity | Aiken’s V range | Assessment items | 0.88–0.96 | Range across item-level coefficients. |
| Internal consistency | Cronbach’s alpha | Assessment battery | 0.83 | Internal consistency estimate for the reported battery. |
| Inter-rater agreement | ICC; two-way random-effects, absolute agreement | Oral expression | 0.89 (95% CI: 0.78–0.95) | Independent ratings by two trained assessors. |
| Inter-rater agreement | ICC; two-way random-effects, absolute agreement | Written expression | 0.91 (95% CI: 0.82–0.96) | Independent ratings by two trained assessors. |
